# Association between sugar-sweetened beverage consumption and childhood overweight and obesity: a cross-sectional study in Beijing, China

**DOI:** 10.3389/fnut.2025.1686320

**Published:** 2025-11-03

**Authors:** Yiran Li, Lulu Meng, Yan Zhang, Wenjia Li, Siyu Liang, Jiarui Zheng, Jie Li, Ruoxiang Cao, Jiali Duan, Liyu Huang

**Affiliations:** ^1^School of Public Health, Hebei Medical University, Shijiazhuang, Hebei, China; ^2^Beijing Center for Disease Prevention and Control, Office of Research and Teaching Administration, Beijing, China; ^3^Fengtai District Center for Disease Prevention and Control, Institute of Nutrition and Food Hygiene, Beijing, China; ^4^School of Public Health, Capital Medical University, Beijing, China; ^5^Beijing Center for Disease Prevention and Control, Institute of Nutrition and Food Hygiene, Beijing, China

**Keywords:** sugar-sweetened beverage, overweight/obesity, consumption, school age, behavior

## Abstract

**Background:**

The consumption of sugar-sweetened beverage (SSB) has significantly increased and is regarded as a potential cause of overweight and obesity in children and adolescents. This study explores the association between SSB consumption and overweight/obesity among primary and secondary school students in Beijing in 2024.

**Methods:**

A total of 3,402 participants were included in this cross-sectional study conducted from October to November 2024. Binary logistic regression is used to assess the association between SSB consumption and weight/obesity.

**Results:**

Approximately 38.5% of the study population were overweight/obese. After adjusting for covariates, SSB consumption was associated with overweight /obesity. Participants with” SSB consumption frequency ≥7 times/week” had a higher prevalence of overweight/obesity compared to participants” SSB consumption frequency < once/week” (OR = 1.396, 95%CI = 1.093–1.782). Participants with” SSB consumption quantity ≥500 mL/day” had a higher prevalence of overweight/obesity compared to those with “SSB consumption quantity <150 mL/day” (OR = 1.316, 95%CI = 1.093–1.586). Moreover, when subgroup analyses were conducted, the association between SSB consumption and overweight/obesity was more susceptible among females, younger students, and urban residents.

**Conclusion:**

The consumption rate of sugar-sweetened beverages among school-age children in Beijing is high. Student sugar-sweetened beverage consumption was significantly associated with overweight/obesity. Therefore, targeted actions and plans should be taken to reduce the consumption of SSB and control childhood obesity.

## 1 Introduction

In recent decades, the global obesity rate among children and adolescents has shown a rapid increase. Between 1990 and 2021, the combined prevalence of overweight and obesity in children and adolescents doubled, and that of obesity alone tripled. Notably, the most significant increase was observed in southeast Asia, east Asia, and Oceania (e.g., Taiwan [province of China], Maldives, and China) (1). The obesity rate among children aged 2–19 years in the United States increased from 14.7% in 1999–19.2% in 2018 (2). Data from the national school health examination in Korea indicated that the obesity prevalence among children aged 6–18 increased from 8.7 to 15.0% between 2007 and 2017 (3). Although obesity was once common in developed countries and regions, with the development of China’s economy and the change of dietary structure, obesity has become one of the public health challenges in China (4, 5). From 2015 to 2019, the results of chronic disease and nutrition monitoring among Chinese residents showed that the overweight and obesity rate of children and adolescents aged 6–17 reached 19% (6). In a cross-sectional survey that included 201,098 children aged 3–18 years, the highest obesity prevalence was estimated for children aged 8–13 years in northern China (from 18.8 to 23.6%) (7). Overweight and obesity are established risk factors for major non-communicable diseases, including cardiovascular diseases, type 2 diabetes, and cancer, which impose significant costs on health, society, and the economy (8–12).

The diet and nutrition in China have undergone profound changes, gradually shifting from a traditional diet of coarse grains and vegetables to a Western-style diet with increased consumption of high-sugar and high-fat foods (5). Sugar-sweetened beverages (SSBs) is the major source of added sugars in the diet (13). Numerous studies have indicated that the consumption of sugary beverages has become one of the significant risk factors contributing to overweight and obesity (14–16). The growing trend of sugary beverage consumption among children and adolescents has become a significant public health concern. A population-based study showed a 23% increase in sugar-sweetened beverage intake among children and adolescents aged 3–19 years in 185 countries from 1990 to 2018, parallel to the rise in obesity prevalence globally (17). A survey in New Zealand showed that 96% of children aged 8–12 consumed at least one serving of sugary drinks per week, and 62% of the children reported consuming five or more servings (18). Results from the Nutrition and Health Monitoring in China conducted from 2010 to 2013 indicated that the estimated intake of SSBs among children aged 6–17 years is 181.0 grams per day, occurring 2.2 times per week (19). However, data specific to Chinese school-aged children remain limited, especially recent regional studies in northern China.

While many studies have reported that higher SSB intake is associated with greater odds of overweight and obesity, the strength and patterns of this association may vary by demographic and environmental contexts. For instance, gender, age, and urban–rural differences may modify the relationship between SSB consumption and body weight. Understanding these differences is important for designing more targeted interventions.

Therefore, this cross-sectional study aimed to (1) describe SSB consumption patterns among primary and secondary school students in Beijing, (2) examine the association between SSB consumption and overweight/obesity, and (3) explore whether this association differs by gender, grade, and residential area. The findings provide localized evidence for designing tailored interventions to reduce SSB intake and prevent childhood obesity in urban China.

## 2 Materials and Methods

### 2.1 Study population

This cross-sectional study was conducted from October to November 2024 by applying a multi stage cluster sampling method to selected 3,402 participants. This investigation was conducted in Beijing, China, selecting three project sites categorized into three areas (central urban areas, outer urban areas, and suburban areas) based on administrative divisions and geographical locations. Two primary schools, two middle schools, and two high schools were chosen from each project site, with participants consisting of third-grade, seventh-grade, and tenth-grade students.

The study was approved by the Ethics Committee of the Beijing Center for Disease Prevention and Control (BJCDC2024031). Written informed consent was obtain from both the student’s caregivers and participating students prior to the study.

### 2.2 Data collection

#### 2.2.1 Childhood overweight/obesity

Height and weight were measured during the semester’s routine physical examination conducted by trained school health professionals following standardized procedures. The data were provided to the research team by the schools. Body mass index (BMI) was calculated as weight (kg) / height (m^2^). Overweight and obesity were defined using the *Screening for Overweight and Obesity among School-Age Children and Adolescents* (WS/T 586–2018) issued by the National Health Commission of China (20). This national reference is the official standard used in China’s child health surveillance and is based on large-scale, population-specific growth data reflecting the physical development characteristics of Chinese children and adolescents.

#### 2.2.2 SSB consumption

SSB consumption was derived from the Student Sugar-Sweetened Beverage Questionnaire, which was developed based on the BEVQ-15. The Cronbach’s *α* coefficient is 0.817, indicating good internal consistency. Each participant was asked to recall the frequency and quantity of sugary drinks consumed over the past week. Both pre-packaged beverages and freshly prepared beverages are included, with pre-packaged beverages consisting of 100% fruit/vegetable juices, fruit/vegetable beverages, carbonated beverages, tea beverages, milk beverages, plant protein drinks, beverages for special uses, coffee beverages.

To assess the weekly consumption of beverages, the questionnaire sets “Drinking Frequency” and “Average Drinking Per Consumption.” “Drinking frequency” is set to “times/day” (drinking once or more a day is reported here), “times/week” (drinking at least once a week, but not consuming every day is reported here), “never” (not drinking in the past week is reported here). Students were asked to report the frequency and quantity of each drink according to their actual situation, and the quantity of drinking measured in milliliter. Finally, the frequency of each drink is unified as “times/week.” Consuming SSB once or more in the past week is defined as having SSB consumption behaviors. SSB consumption frequency was divided into three categories: “<once/week,” “1 to <7 times/week,” and “≥7 times/week.” SSB consumption quantity was also divided into three categories: “<150 mL/day,” “150 to <500 mL/day,” and “≥500 mL/day.”

#### 2.2.3 Confounding variables

Covariates were selected based on prior literature and the availability of data in this survey. They included demographic variables and health-related behavior factors.

Demographic characteristics included: gender, age, grade, residence, and caregiver education level. The educational stages were divided into primary school (3th), middle school (7th), and high school (10th). Residence was categorized as central urban areas, outer urban areas, and suburban areas. The caregiver education level was classified as below bachelor’s degree, bachelor’s degree and above.

Health-related behaviors included: daily water intake (<1,000 mL/ day, 1,000 to <1,500 mL/ day, and ≥1,500 mL/ day), daily beverage charge (<10 yuan/ day, and ≥10 yuan/ day), and daily outdoor activity time (<60 min/ day and ≥60 min/ day).

### 2.3 Statistical analyses

All statistical analyses were performed using the statistical software IBM SPSS Statistics version 22.0. Descriptive analysis of demographic information and health-related behaviors on categorical variables was conducted utilizing numbers and proportions, while differences between these characteristic variables and weight status and frequency and quantity of SSB consumption were verified by chi-square test. To assess the independent association of SSB consumption with overweight/obesity, binary logistic regression was performed. Model 1 was adjusted for gender and grade. Model 2 was adjusted for gender, grade, residence and caregiver education level. Model 3 was adjusted for gender, grade, residence, caregiver education level and health-related behaviors. The stratification variable was removed from the model in order to conduct a further analysis of gender, grade and residence subgroup stratification using the same modeling approach as previously mentioned. All statistical significance tests were two-sided, and a level of *α* < 0.05 was considered statistically significant.

## 3 Results

### 3.1 Demographic characteristics

A total of 3,402 children were included in this study, ranging in age range from 7 to 17 years old. The proportions of male and female children were almost equal. The number of students in different grades was balanced. The majority of caregivers (62.8%) had a bachelor’s degree or higher education level. Only a small percentage of students (24.1%) drunk less than 1,000 mL of water per day. Most students (63.8%) spent less than 10 yuan per day on drinks. More than half of the students (52.0%) were able to spend 60 min or more outdoors activity per day.

The overall prevalence of overweight/obesity was 38.5%. Students who were male, in higher grades, residing in suburban areas, whose caregiver had a low level of education, and who consumed an average daily water intake of more than 1,500 mL were at high risk of being overweight and obese (*Ρ* < 0.05) (Table 1).

**Table 1:** The demographic characteristics of 3,402 students based on body weight status in Beijing.

Characteristics	N (%)	Overweight/obesity, *n* (%)^a^			
		No	Yes	*χ* ^2^	*P*-value
Total	3,402 (100.0)	2092 (61.5)	1,310 (38.5)		
Gender				153.405	*Ρ* < 0.001
Male	1712 (50.3)	877 (51.2)	835 (48.8)		
Female	1,690 (49.7)	1,215 (71.9)	475 (28.1)		
Grade				21.758	*Ρ* < 0.001
Primary school (3th)	1,090 (32.0)	732 (67.2)	358 (32.8)		
Middle school (7th)	1,164 (34.2)	687 (59.0)	477 (41.0)		
High school (10th)	1,148 (33.7)	673 (58.6)	475 (41.4)		
Residence				14.892	*Ρ* < 0.001
Central urban areas	1,177 (34.6)	774 (65.8)	403 (34.2)		
Outer urban areas	1,069 (31.4)	645 (60.3)	424 (39.7)		
Suburban areas	1,156 (34.0)	673 (58.2)	483 (41.8)		
Caregiver education level				11.384	*Ρ* < 0.001
Below bachelor’s degree	1,264 (37.2)	731 (57.8)	533 (42.2)		
Bachelor’s degree and above	2,138 (62.8)	1,361 (63.7)	777 (36.3)		
Daily water intake				36.681	*Ρ* < 0.001
<1,000 mL/day	819 (24.1)	557 (68.0)	262 (32.0)		
1,000 to <1,500 mL/day	1,139 (33.5)	728 (63.9)	411 (36.1)		
≥1,500 mL/day	1,444 (42.4)	807 (55.9)	637 (44.1)		
Daily beverage charge				0.036	0.849
<10 yuan/day	2,170 (63.8)	1,337 (61.6)	833 (38.4)		
≥10 yuan/day	1,232 (36.2)	755 (61.3)	477 (38.7)		
Daily outdoor activity time				0.191	0.662
<60 min/day	1,634 (48.0)	1,011 (61.9)	623 (38.1)		
≥60 min/day	1768 (52.0)	1,081 (61.1)	687 (38.9)		

^a^Weighted percentage may not add up to 100% due to rounding.

### 3.2 Frequency of SSB consumption

Table 2 shows the frequency of SSB consumption for all children by gender, grade, residence, caregiver education level, daily water intake, daily beverage charge, and daily outdoor activity time. Overall, the consumption rate of SSB among school-age children in Beijing was 88.4%. About 11.6% of children consumed SSB < once/week, and 57.1% of children consumed SSB ≥ 7 times/week.

**Table 2:** The frequency of SSB consumption among participants in different groups in Beijing.

Characteristics	Frequency of SSB consumption, *n* (%)^a^			*χ* ^2^	*P*-value
	<once/week	1 to <7 times/week	≥7 times/week		
Total	394 (11.6)	1,067 (31.4)	1941 (57.1)		
Gender				0.077	0.962
Male	196 (11.4)	536 (31.3)	980 (57.2)		
Female	198 (11.7)	531 (31.4)	961 (56.9)		
Grade				151.736	*Ρ* < 0.001
Primary school (3th)	198 (18.2)	424 (38.9)	468 (42.9)		
Middle school (7th)	108 (9.3)	345 (29.6)	711 (61.1)		
High school (10th)	88 (7.7)	298 (26.0)	762 (66.4)		
Residence				27.119	*Ρ* < 0.001
Central urban areas	145 (12.3)	415 (35.3)	617 (52.4)		
Outer urban areas	116 (10.9)	349 (32.6)	604 (56.5)		
Suburban areas	133 (11.5)	303 (26.2)	720 (62.3)		
Caregiver education level				15.760	*Ρ* < 0.001
Below bachelor’s degree	127 (10.0)	361 (28.6)	776 (61.4)		
Bachelor’s degree and above	267 (12.5)	706 (33.0)	1,165 (54.5)		
Daily water intake				27.003	*Ρ* < 0.001
<1,000 mL/day	113 (13.8)	291 (35.5)	415 (50.7)		
1,000 to <1,500 mL/day	135 (11.9)	368 (32.3)	636 (55.8)		
≥1,500 mL/ day	146 (10.1)	408 (28.3)	890 (61.6)		
Daily beverage charge				226.254	*Ρ* < 0.001
<10 yuan/ day	317 (14.6)	823 (37.9)	1,030 (47.5)		
≥10 yuan/ day	77 (6.3)	244 (19.8)	911 (73.9)		
Daily outdoor activity time				0.114	0.944
<60 min/day	188 (11.5)	517 (31.6)	929 (56.9)		
≥60 min/day	206 (11.7)	550 (31.1)	1,012 (57.2)		

^a^Weighted percentage may not add up to 100% due to rounding.

The proportion of students in higher grades, residing in suburban areas, with low caregiver education levels, whose daily water intake ≥1,500 mL/ day, and daily beverage charge ≥10 yuan/day, was significantly higher for those consuming sugary drinks ≥7 times/week (*Ρ* < 0.05).

### 3.3 Quantity of SSB consumption

Table 3 shows the frequency of SSB consumption for all children by gender, grade, residence, caregiver education level, daily water intake, daily beverage charge, and daily outdoor activity time. The proportion of students who were males, senior students, living in the suburbs, with low caregiver education levels, whose daily water intake ≥1,500 mL/day, and daily beverage ≥10 yuan/day had a higher proportion of SSB consumption quantity ≥500 mL/day (*Ρ* < 0.05).

**Table 3:** The quantity of SSB consumption among participants in different groups in Beijing.

Characteristics	Quantity of SSB consumption, *n* (%)^a^			*χ* ^2^	*P*-value
	<150 mL/day	150 to <500 mL/day	≥500 mL/day		
Total	1,096(32.2)	1,019 (30.0)	1,287 (37.8)		
Gender				11.324	0.003
Male	531 (31.0)	486 (28.4)	695 (40.6)		
Female	565 (33.4)	533 (31.5)	592 (35.0)		
Grade				292.205	*Ρ* < 0.001
Primary school (3th)	557 (51.1)	283 (26.0)	250 (22.9)		
Middle school (7th)	309 (26.5)	363 (31.2)	492 (42.3)		
High school (10th)	230 (20.0)	373 (32.5)	545 (47.5)		
Residence				30.077	*Ρ* < 0.001
Central urban areas	417 (35.4)	375 (31.9)	385 (32.7)		
Outer urban areas	356 (33.3)	311 (29.1)	402 (37.6)		
Suburban areas	323 (27.9)	333 (28.8)	500 (43.3)		
Caregiver education level				21.019	*Ρ* < 0.001
Below bachelor’s degree	350 (27.7)	387 (30.6)	527 (41.7)		
Bachelor’s degree and above	746 (34.9)	632 (29.6)	760 (35.5)		
Daily water intake				31.301	*Ρ* < 0.001
<1,000 mL/day	316 (38.6)	239 (29.2)	264 (32.2)		
1,000 to <1,500 mL/day	378 (33.2)	334 (29.3)	427 (37.5)		
≥1,500 mL/ day	402 (27.8)	446 (30.9)	596 (41.3)		
Daily beverage charge				262.271	*Ρ* < 0.001
<10 yuan/day	880 (40.6)	667 (30.7)	623 (28.7)		
≥10 yuan/day	216 (17.5)	352 (28.6)	664 (53.9)		
Daily outdoor activity time				0.277	0.871
<60 min/day	533 (32.6)	489 (29.9)	612 (37.5)		
≥60 min/day	563 (31.8)	530 (30.0)	675 (38.2)		

^a^Weighted percentage may not add up to 100% due to rounding.

### 3.4 SSB consumption and overweight/obesity

As shown in Table 4, participants who reported consuming SSB ≥ 7 times/week showed a higher prevalence of overweight/obese compared to participants who consumed SSB < once/week (Model 1: OR = 1.400,95%CI = 1.102–1.779; Model 2: OR = 1.380, 95%CI = 1.086–1.755; Model 3: OR = 1.396, 95%CI = 1.093–1.782). Participants who reported consuming SSB ≥ 500 mL/day showed a higher prevalence of overweight/obese compared to those consuming SSB < 150 mL/day (Model 1: OR = 1.334,95%CI = 1.116–1.595; Model 2: OR = 1.295, 95%CI = 1.082–1.549; Model 3: OR = 1.316, 95%CI = 1.093–1.586).

**Table 4:** Association of SSB consumption with overweight/obesity in the total study population.

SSB consumption	Model 1^a^		Model 2^b^		Model 3^c^	
	*OR* (95%*CI*)	*P*-value	OR (95%CI)	*P*-value	OR (95%CI)	*P*-value
Frequency						
<Once/week	Ref.		Ref.		Ref.	
1 to <7 times/week	1.140(0.886 ~ 1.467)	0.310	1.154(0.896 ~ 1.488)	0.267	1.163(0.901 ~ 1.500)	0.247
≥7 times/week	1.400(1.102 ~ 1.779)	0.006**	1.380(1.086 ~ 1.755)	0.008**	1.396(1.093 ~ 1.782)	0.008**
Quantity						
<150 mL/day	Ref.		Ref.		Ref.	
150 to <500 mL/day	1.133(0.940 ~ 1.364)	0.190	1.127(0.935 ~ 1.358)	0.210	1.132(0.937 ~ 1.367)	0.198
≥500 mL/day	1.334(1.116 ~ 1.595)	0.002**	1.295(1.082 ~ 1.549)	0.005**	1.316(1.093 ~ 1.586)	0.004**

^a^Gender variable and grade variable were controlled for in Model 1. ^b^Based on Model 1, Model 2 also controlled for residence, and caregiver education level. ^c^Based on Model 2, Model 3 also controlled for health-related behaviors. Ref. indicates reference category. OR, odds ratio; CI, confidence interval. **Ρ < 0.001.

### 3.5 Subgroup analysis of gender, grade, and residence stratification

In the gender subgroup analysis, three models showed that the statistically significant associations of SSB consumption frequency-overweight/obesity were observed only in females, in which a similar result was found for SSB consumption quantity-overweight/obesity. In contrast to SSB consumption frequency <once/week, SSB consumption frequency ≥7 times/week was associated with higher odds of overweight or obese among females (Model 1: OR = 1.525, 95% CI = 1.055–2.204; Model 2: OR = 1,491, 95% CI = 1.031–2.158; Model 3: OR = 1.509, 95% CI = 1.035–2.201). As opposed to females who had SSB consumption quantity <150 mL/day, those who had SSB consumption quantity 150 to <500 mL/day (Model 1 of Table 5: OR = 1.336, 95% CI = 1.016–1.758; Model 3 of Table 5: OR = 1.324, 95% CI = 1.003–1.748) and SSB consumption quantity ≥500 mL/day (Model 1 of Table 5: OR = 1.423, 95% CI = 1.087–1.863; Model 2 of Table 5: OR = 1.368, 95% CI = 1.043–1.794; Model 3 of Table 5: OR = 1.416, 95% CI = 1.067–1.880) were associated with higher odds of overweight or obese (Table 5).

**Table 5:** Association of SSBs consumption with overweight/obesity by gender subgroups.

SSBs consumption		By gender group	
		Male OR (95%CI)	Female OR (95%CI)
Frequency (reference group is “<once/week”)			
Model 1^a^	1 to <7 times/week	1.040 (0.745 ~ 1.452)	1.276 (0.864 ~ 1.885)
	≥7 times/week	1.300 (0.946 ~ 1.787)	1.525 (1.055 ~ 2.204) *
Model 2^b^	1 to <7 times/week	1.045 (0.746 ~ 1.463)	1.291 (0.873 ~ 1.910)
	≥7 times/week	1.280 (0.930 ~ 1.762)	1.491 (1.031 ~ 2.158) *
Model 3^c^	1 to <7 times/week	1.075 (0.766 ~ 1.509)	1.288 (0.870 ~ 1.906)
	≥7 times/week	1.320 (0.952 ~ 1.828)	1.509 (1.035 ~ 2.201) *
Quantity (reference group is “<150 mL/day”)			
Model 1^a^	150 to <500 mL/day	0.981 (0.760 ~ 1.264)	1.336 (1.016 ~ 1.758) *
	≥500 mL/day	1.250 (0.983 ~ 1.589)	1.423 (1.087 ~ 1.863) *
Model 2^b^	150 to <500 mL/day	0.984 (0.762 ~ 1.270)	1.311 (0.995 ~ 1.727)
	≥500 mL/day	1.218 (0.956 ~ 1.551)	1.368 (1.043 ~ 1.794) *
Model 3^c^	150 to <500 mL/day	0.987 (0.762 ~ 1.279)	1.324 (1.003 ~ 1.748) *
	≥500 mL/day	1.237 (0.963 ~ 1.590)	1.416 (1.067 ~ 1.880) *

^a^Gender variable and grade variable were controlled for in Model 1. ^b^Based on Model 1, Model 2 also controlled for residence, and caregiver education level. ^c^Based on Model 2, Model 3 also controlled for health-related behaviors. Ref. indicates reference category. OR, odds ratio; CI, confidence interval. *Ρ < 0.05.

In the grade subgroup analysis, model 1 showed that the statistically significant association of SSB consumption frequency-overweight/obesity was observed only in primary school (3th), in which a similar result was found for SSB consumption quantity-overweight/obesity. Primary school students with SSB consumption frequency ≥7 times/week were associated with higher odds of overweight or obese than those who consumed SSB < once/week (Model 1: OR = 1.564, 95%CI = 1.080–2.264). Primary school students who consumed 150 to <500 mL/day and ≥500 mL/day were associated with higher odds of overweight or obese than SSB consumption < 150 mL/day (Model 1: OR = 1.368, 95%CI = 1.005–1.861; OR = 1.520, 95%CI = 1.106–2.090) (Table 6).

**Table 6:** Association of SSBs consumption with overweight/obesity by grade subgroups.

SSBs consumption		By grade group		
		Primary school (3th) OR (95%CI)	Middle school (7th) OR (95%CI)	High school (10th) OR (95%CI)
Frequency (reference group is “<once/week”)				
Model 1^a^	1 to <7 times/week	1.268 (0.869 ~ 1.851)	0.992 (0.627 ~ 1.570)	1.080 (0.649 ~ 1.798)
	≥7 times/week	1.564 (1.080 ~ 2.264) *	1.204 (0.784 ~ 1.848)	1.347 (0.839 ~ 2.162)
Model 2^b^	1 to <7 times/week	1.262 (0.863 ~ 1.844)	1.022 (0.644 ~ 1.623)	1.149 (0.687 ~ 1.922)
	≥7 times/week	1.449 (0.992 ~ 2.118)	1.253 (0.813 ~ 1.931)	1.399 (0.869 ~ 2.252)
Model 3^c^	1 to <7 times/week	1.256 (0.859 ~ 1.838)	1.051 (0.659 ~ 1.676)	1.165 (0.694 ~ 1.953)
	≥7 times/week	1.469 (0.997 ~ 2.165)	1.273 (0.817 ~ 1.983)	1.430 (0.882 ~ 2.316)
Quantity (reference group is “<150 mL/day”)				
Model 1^a^	150 to <500 mL/day	1.368 (1.005 ~ 1.861) *	0.788 (0.570 ~ 1.089)	1.299 (0.917 ~ 1.839)
	≥500 mL/day	1.520 (1.106 ~ 2.090) *	1.194 (0.886 ~ 1.609)	1.263 (0.911 ~ 1.750)
Model 2^b^	150 to <500 mL/day	1.336 (0.978 ~ 1.825)	0.810 (0.585 ~ 1.122)	1.324 (0.933 ~ 1.879)
	≥500 mL/day	1.386(0.986 ~ 1.948)	1.206 (0.892 ~ 1.630)	1.274 (0.917 ~ 1.768)
Model 3^c^	150 to <500 mL/day	1.367 (0.997 ~ 1.875)	0.819 (0.589 ~ 1.138)	1.323 (0.930 ~ 1.881)
	≥500 mL/day	1.428 (1.000 ~ 2.040)	1.203 (0.879 ~ 1.647)	1.300 (0.929 ~ 1.820)

^a^Gender variable and grade variable were controlled for in Model 1. ^b^Based on Model 1, Model 2 also controlled for residence, and caregiver education level. ^c^Based on Model 2, Model 3 also controlled for health-related behaviors. Ref. indicates reference category. OR, odds ratio; CI, confidence interval. *Ρ < 0.05.

In the residence subgroup analysis, three models showed that the statistically significant associations of SSB consumption quantity-overweight/obesity were observed only in central urban areas. Participants in central urban areas who reported consuming SSB ≥ 500 mL/day were associated with higher odds of overweight or obese compared to those consuming SSB < 150 mL/day (Model 1: OR = 1.129, 95% CI = 1.021–2.001; Model 2: OR = 1.428, 95% CI = 1.020–1.998 and Model 3: OR = 1.419, 95% CI = 1.005–2.004) (Table 7).

**Table 7:** Association of SSBs consumption with overweight/obesity by residence subgroups.

SSBs consumption		By residence group		
		Central urban areas OR (95%CI)	Outer urban areas OR (95%CI)	Suburban areas OR (95%CI)
Frequency (reference group is “<once/week”)				
Model 1^a^	1 to <7 times/week	0.909 (0.593 ~ 1.395)	1.404 (0.881 ~ 2.236)	1.255 (0.815 ~ 1.933)
	≥7 times/week	1.264 (0.830 ~ 1.925)	1.496 (0.954 ~ 2.346)	1.438 (0.970 ~ 2.132)
Model 2^b^	1 to <7 times/week	0.909 (0.593 ~ 1.395)	1.401 (0.879 ~ 2.232)	1.255 (0.814 ~ 1.934)
	≥7 times/week	1.263 (0.830 ~ 1.924)	1.486 (0.947 ~ 2.331)	1.441 (0.972 ~ 2.138)
Model 3^c^	1 to <7 times/week	0.901(0.587 ~ 1.384)	1.385 (0.868 ~ 2.209)	1.298 (0.837 ~ 2.014)
	≥7 times/week	1.247 (0.814 ~ 1.912)	1.555 (0.984 ~ 2.457)	1.444 (0.963 ~ 2.165)
Quantity (reference group is “<150 mL/day”)				
Model 1^a^	150 to <500 mL/day	1.279 (0.929 ~ 1.763)	1.034 (0.739 ~ 1.445)	1.076 (0.779 ~ 1.486)
	≥500 mL/day	1.129 (1.021 ~ 2.001) *	1.142 (0.827 ~ 1.579)	1.331 (0.992 ~ 1.785)
Model 2^b^	150 to <500 mL/day	1.278 (0.928 ~ 1.761)	1.028 (0.735 ~ 1.437)	1.081 (0.782 ~ 1.494)
	≥500 mL/day	1.428 (1.020 ~ 1.998) *	1.138 (0.823 ~ 1.573)	1.332 (0.992 ~ 1.788)
Model 3^c^	150 to <500 mL/day	1.257 (0.908 ~ 1.740)	1.061 (0.756 ~ 1.490)	1.085 (0.781 ~ 1.507)
	≥500 mL/day	1.419 (1.005 ~ 2.004) *	1.225 (0.875 ~ 1.715)	1.316 (0.969 ~ 1.788)

^a^Gender variable and grade variable were controlled for in Model 1. ^b^Based on Model 1, Model 2 also controlled for residence, and caregiver education level. ^c^Based on Model 2, Model 3 also controlled for health-related behaviors. Ref. indicates reference category. OR, odds ratio; CI, confidence interval. *Ρ < 0.05.

## 4 Discussion

This study investigated the consumption of sugar-sweetened beverages among primary and secondary school students in Beijing and its association with overweight and obesity. The results indicated that 88.5% of school-age children had consumed SSBs, which is higher than the consumption rate among young children in China in 2016 (21). This is similar to the rate of consumption of sugar-sweetened beverages among children in the cohort study of the 2004–2011 China Health and Nutrition Survey (87.6%), and lower than that of children aged 8–12 years in New Zealand (96.0%) (18).

Our study showed that 57.1% of students consume sugary beverages ≥7 times a week, with an average daily consumption of ≥500 mL accounting for 37.8%. Male students, those in higher grades, are more likely to consume sugary beverages, which is consistent with previous research findings (19). Unlike previous studies (19, 22), this research indicates that children in suburban areas have a higher consumption rate of sugary beverages compared to those in urban areas, which may be related to the lower socio-economic status or higher density of construction in the region (23). In addition, low education levels of caregivers, excessive daily water intake, and high expenditure on beverages are all associated with high consumption of sugary drinks. The increase in sugary drink consumption among children is related to multiple factors, primarily including demographic characteristics, family factors, socioeconomic status, and so on (24–27). In order to curb the growing trend of sugary beverage consumption, many countries and regions have implemented sugar reduction measures (28, 29), among which sugar taxation serves as a significant policy intervention aimed at improving nutrition and public health (30).

The overweight and obesity rate among children and adolescents in Beijing is 38.5%, which is consistent with the overweight and obesity rate of 38.2% reported in a survey conducted in Jiangsu Province, China, involving 119,467 students aged 8–17 years (31). Our research found that children who consume SSBs at higher frequencies and quantities are more likely to be overweight and obese. After adjusting for demographic and health-related behavioral confounders, children who consumed SSBs ≥7 times/week” were more likely to be overweight/obese compared to who consumed SSBs <once/week (OR = 1.396, 95%CI = 1.093–1.782). children who consumed SSBs ≥500 mL/day” had a higher risk of being overweight/obese than children who consumed SSBs <150 mL/day.” (OR = 1.316, 95%CI = 1.093–1.586). From a biological perspective, several mechanisms may explain these associations. Liquid calories from SSBs are less satiating than solid foods, leading to incomplete energy compensation at subsequent meals. Frequent SSB consumption induces sharp fluctuations in blood glucose and insulin levels, promoting fat deposition and insulin resistance. Fructose, in particular, enhances hepatic *de novo* lipogenesis and reduces leptin signaling, resulting in increased appetite and energy storage. These physiological mechanisms support the consistent finding that frequent SSB consumption contributes to energy imbalance and adiposity accumulation in children.

Previous studies have reported similar associations in other regions. Research by Marshall et al. indicated that each additional 8 oz. SSB consumed/day throughout childhood and adolescence increased the BMI z score an average 0.050 units (95%CI = 0.022–0.079; *Ρ* = 0.001) (15). The nationwide cross-sectional analysis of Chinese children and adolescents aged 6 to 17 in 2014 showed that participants who were high consumers of SSB had a higher odds ratio (OR = 1.133, 95%CI = 1.054–1.217) for having abdominal obesity compared to non-consumers (32). Research findings from South China showed that, in contrast to non-consumers, the adjusted odds ratio of consuming SSBs more than 120 mL/day is 2.08 (95%CI = 1.21–3.54) for obesity and 1.83 (95%CI = 1.25–2.69) for abdominal obesity among consumers (33). The Singapore GUSTO mother-offspring cohort indicated that SSB intake in young childhood is associated with higher risks of adiposity and overweight/obesity (34). However, our research findings differ from those of Zhang Ting et al. The data from the ‘Children of 1997’ birth cohort revealed that neither the frequency of SSB consumption at ages 11 nor 13 was associated with subsequent BMI z-scores or overweight/obesity up to age 18 (35). The Korean Child-Adolescent Cohort Study showed that there were no significant associations between total sugar intake and adiposity and continuous metabolic syndrome scores (36). These inconsistencies may be attributed to factors such as heterogeneity across different regions and ethnicities, as well as variations in the sample size of the studies.

In addition, this paper evaluated the association of gender, grade and residence with SSB and overweight or obesity. It has been observed that the higher the frequency and quantity of SSB consumption among female students, the more likely it is to be overweight/obese. Similarly, SSB high consumption was found to be associated with higher body fat percentage (BF%) in females in New Zealand school-age children (18). A potential explanation being that female students seemed to be more prone to SSB consumption alone or in combination with inadequate moderate-to-vigorous physical activity than their counterparts (31), which may be an important reason for the gender differences in the association of SSB with obesity. Socio-behavioral mechanisms may further explain subgroup differences. The stronger association between SSB intake and obesity among female students may relate to gendered patterns of beverage preference and emotional eating. Girls are often more sensitive to body image pressures and may alternate between dietary restraint and stress-related consumption of high-sugar drinks. In contrast, male students’ higher baseline SSB intake may reflect greater energy requirements or peer-influenced behaviors. For urban–rural differences, students in central urban areas showed a more pronounced link between SSB intake and obesity, possibly due to greater access to commercial beverages, targeted marketing in cities, and reduced outdoor physical activity opportunities. Data from Indonesia showed similar urban trends, where that daily consumption of caffeinated soft drinks and energy drinks was positively associated with overweight and obesity in urban areas (OR = 1.12, 95%CI = 1.01–1.23) (37). These observations underscore the role of environmental exposure and socioeconomic context in shaping dietary behaviors, aligning with the social–ecological model of health behavior. Age-related variations may reflect developmental and psychosocial differences. Younger students are more influenced by parental purchasing and home availability, while older adolescents have greater autonomy and exposure to peer norms and media marketing.

Although the associations identified in this study were statistically significant, the effect sizes were relatively modest (ORs ≈ 1.3–1.4). These findings are consistent with other population-based studies, such as the 2014 national survey in China (OR = 1.13 for abdominal obesity) (32). The relatively modest magnitude observed in our study may be attributed to several factors, including regional dietary differences, limited variation in SSB intake among Beijing students, and the potential influence of unmeasured confounders such as total energy intake, overall dietary pattern, and parental BMI. Inconsistencies with prior studies reporting stronger associations (e.g., ORs ≈ 1.8–2.1 in South China) (33) may reflect heterogeneity in cultural, environmental, and socioeconomic contexts. Southern regions of China typically experience higher temperatures and greater availability of sweetened beverages, while children there may also have higher outdoor activity levels and fluid replacement needs, influencing consumption patterns. Moreover, studies using different definitions of SSBs or obesity criteria (e.g., WHO vs. Chinese national standards) may yield varying prevalence and effect estimates. Despite the modest ORs, these associations have substantial public health implications. Given the high prevalence of SSB consumption, even small individual effects can translate into a large population-level burden of overweight and obesity. Therefore, the findings emphasize the importance of population-based interventions to reduce SSB intake as part of comprehensive childhood obesity prevention strategies.

Frequent excessive consumption of sugary beverages not only triggers obesity but also serves as a risk factor for various diseases such as type 2 diabetes, cardiovascular diseases, and dental caries (38, 39). Therefore, the World Health Organization recommends the implementation of detailed measures to reduce the excessive intake of sugary beverages among children and adolescents. Currently, more than 45 countries and regions have implemented sugar tax policies (40). Various interventions such as nutrition labeling (41, 42), advertising regulation (43, 44), economic tools (45), and interventions in families and schools have been put into practice (46, 47), which have been proven effective in reducing the consumption of sugar-sweetened beverages. Beyond taxation, community-based and school-level health education should also emphasize emotional regulation, body image awareness, and media literacy, given the sociopsychological factors influencing beverage choices among youth.

This study has some strengths and limitations. It provides recent, population-based evidence on SSB consumption and obesity among Beijing students and explores subgroup differences by gender, grade, and residence, which are valuable for targeted intervention design. Nevertheless, the cross-sectional design precludes causal inference. Self-reported SSB intake may involve recall and social desirability bias. Second, the SSB consumption data is self-reported and may be subject to recall bias. In addition, this study describes the relationship between SSB consumption behavior and obesity among children in Beijing, and the results cannot be generalized to the whole population. The present study used the Chinese national BMI reference (WS/T 586–2018) to define overweight and obesity, which is most appropriate for local growth monitoring. However, the prevalence estimates might differ slightly if the WHO BMI-for-age reference were applied, and direct international comparison should therefore be made with caution. The factors that cause obesity are complex, and this paper focuses on the consumption behavior of sugary drinks, and other confounding factors such as socioeconomic level, dietary structure, parental weight, etc. can be further discussed in future studies. Future longitudinal studies combining dietary records, biomarkers, and family-level factors are needed to clarify causal pathways and identify modifiable targets for intervention.

## 5 Conclusion

In conclusion, this study showed that SSB were commonly consumed among school-age children in Beijing. Children who were male, in higher grades, from suburban areas, and with a low level of caregiver education tended to consume SSB more frequently. Children with higher frequency and greater quantity of SSB consumption were significantly associated with overweight or obesity. This association appeared stronger among females, younger students, and children living in central urban areas. Given the growing burden of childhood overweight and obesity, comprehensive interventions are urgently needed to reduce SSB intake among children and adolescents. Such strategies should involve schools, families, and communities, focusing on nutrition education, restriction of sugary drink marketing, and promotion of healthy beverage choices. Future longitudinal and cohort studies are warranted to clarify causal pathways and identify modifiable factors contributing to SSB-related obesity, thereby providing stronger evidence to inform public health policies and prevention programs in China and beyond.

## Data Availability

The original contributions presented in the study are included in the article/supplementary material, further inquiries can be directed to the corresponding authors.
